# Predicting the outcome of normal pressure hydrocephalus therapy—where do we stand?

**DOI:** 10.1007/s00701-020-04700-3

**Published:** 2021-01-20

**Authors:** Joachim M. K. Oertel, Matthias J. M. Huelser

**Affiliations:** Department of Neurosurgery, Saarland University Medical Center and Saarland University Faculty of Medicine, Homburg, Saar Germany

Normal-pressure hydrocephalus (NPH) is a treatable disease. It is the only form of dementia for which we do have effective treatment modalities. Permanent cerebrospinal fluid diversion via shunt insertion is the gold standard. The objective response to the shunt treatment is about 85% [6], at least for a certain time. The majority of patients (85 %) respond to this treatment, but what about the other 15%? How can we predict which patient will respond to treatment and who will not?

Well, the first step consists of being certain about the correct diagnosis, which can be tricky since the pathophysiology of NPH remains not entirely understood. Then, the most challenging aspect is to decide which patient will respond and who will not.

There are plenty of studies investigating the predictors of shunt response.

First of all, what about acquired risk factors? The existence of more than one cardiovascular risk factor seems to be a reliable clinical predictor for a negative outcome in normal pressure hydrocephalus [9]. Solely, age does not account as a risk factor [9, 11].

Commonly, the most important positive predictors for treatment response are considered to be the spinal tab test or continuous lumbar drainage. Even though, the only blinded prospective study on this subject demonstrated a positive predictive value of 88%, but also a negative predictive value of 18% [23]. This results only in an overall accuracy of 53% [6, 23]. Furthermore, continuous lumbar drainage over a few days shows very high positive predictive values, but it has also low negative predictive values [14], so in conclusion, a response to a lumbar drainage test correlates well with a positive response to shunt treatment [8], but a non-response should not exclude the patient from treatment [23], so this nonresponsive patient cohort should be subject to further investigations.

What about radiological findings predicting a treatment response? One study investigated 168 patients with normal pressure hydrocephalus for an association of certain radiological signs, such as disproportionately enlarged subarachnoid space hydrocephalus (DESH) sign, Evans-index, and callosal angle (CA), with the patient outcome after shunt treatment. In this study, no correlation between MRI findings and outcome could be demonstrated [1]. Additionally, the absence or presence of periventricular hyperintensities did not seem to correlate [10].

Although some evidence exists that the DESH sign correlates with a positive response to shunt therapy [7, 22], augmented postischaemic lacunes may imply a rather worse patient outcome [7].

In addition, in a study from 2014, the authors could show that a preoperative steeper CA correlated with a better response to surgery [21].

Similarly, the findings of Mantovani et al. that will be presented in the following demonstrated in their recent study with a statistically significant correlation of the so-called anterior callosal angle (ACA) with an improvement of gait and balance.

Furthermore, three studies with a fair amount of scientific evidence were able to relate a higher aqueduct velocity with positive responsiveness, especially in cases where a cerebrospinal infusion test had been pathological [2, 5, 16].

Furthermore, the reactivity of cerebral blood flow to acetazolamide (measured in prospective study via SPECT) seems to correlate significantly with a response to shunt treatment [3].

Measurement of ICP dynamics is an invasive but useful modality to clarify the question of responsiveness to treatment. Increased outflow resistance during a CSF infusion test seems to be a predictor [13, 19, 23]. Furthermore, pulsatile ICP like pulse pressure amplitudes and vasogenic slow waves can be used for forecasting treatment success [18, 20].

Lastly, there remains the question of whether or not patients with Alzheimer’s disease as comorbidity (up to 19% of cases [17]) to NPH are eligible for shunt treatment? This cannot be answered at this point sufficiently [12, 15]. There is some evidence that phospho-tau as a CSF biomarker seems to have a predictive value for higher postoperative morbidity but on the contrary, there is also data suggesting that patients with comorbid neurodegenerative diseases responded well to shunt treatment [4], but how does this scientific data translate into clinical practice?

In our opinion, the first step is performing a spinal tab test on patients with typical clinical signs and imaging findings for NPH. A special focus should be on the CA, in particular the ACA, when evaluating such as patient imaging. In cases of clinical improvement after spinal tab testing, shunt replacement therapy can be recommended, regardless of supposedly negative predictors. In cases of nonresponsiveness or contraindications for spinal tab testing, further diagnostic investigations such as SPECT imaging, measurement of aqueduct velocity or CSF infusion test, depending on the individual center expertise, may be helpful. In our center, ICP dynamics via telemetric measurement has proven to be a very valuable tool in such cases. In conclusion, despite there being negative predictors for shunt therapy, it is important that one weighs the positive against the negative predictors in individual decision-making for shunt surgery, and that the value of negative predictors should not be overestimated and therefore result in a patient not receiving adequate therapy.
